# Spinopelvic mobility patterns in patients with dislocation after THA: Direct anterior versus posterior approach

**DOI:** 10.1186/s42836-026-00386-7

**Published:** 2026-04-13

**Authors:** Thomas Aubert, Antoine Mouton, Guillaume Auberger, Michael Butnaru, Nicolas Guegan, Christopher Plaskos

**Affiliations:** 1 Groupe Hospitalier Diaconesses Croix Saint Simon, 125 Rue d’Avron, 75020 Paris, France; 2 Corin, Clinical Innovation, Raynham, MA 02767 USA

**Keywords:** Dislocation, Total hip arthroplasty, Spinopelvic mobility, Spine-hip relationship

## Abstract

**Background:**

Dislocation remains a common reason for revision after total hip arthroplasty (THA), and adverse spinopelvic mobility is increasingly recognized as a major contributor to instability. Whether its prevalence differs between surgical approaches is unclear. This study compared adverse spinopelvic mobility and associated risk factors in patients who sustained a dislocation after a posterior approach (PA) or a direct anterior approach (DAA), and evaluated whether implant positioning differed between groups.

**Methods:**

A retrospective analysis was performed on 133 patients with post-operative dislocation and available functional lateral radiographs and low-dose CT scans, including 85 PA and 48 DAA dislocations. Spinopelvic parameters, dynamic pelvic motion between standing, sitting, and supine postures, and established spinopelvic risk factors were assessed. Cup orientation, femoral version, combined anteversion, limb length, and global offset were compared.

**Results:**

Spinopelvic morphology was similar, with no significant differences in standing spinopelvic tilt, pelvic incidence, PI-LL mismatch, lumbar lordosis, or lumbar flexion. However, dynamic motion differed markedly. A change in spinopelvic tilt (SPT) of ≥ 20° from standing to seated occurred in 41.3% of DAA dislocations versus 21.1% after PA (*p* = 0.029). A change in SPT ≤  − 13° from supine to standing occurred in 17.8% after DAA compared with 4.7% after PA (*p* = 0.048). Other spinopelvic risk factors showed no significant differences. Implant positioning was largely comparable; femoral and combined anteversion, cup inclination, and limb length were similar. Although femoral head size and global offset differed between groups, the association between surgical approach and adverse spinopelvic mobility persisted after accounting for these implant-related factors.

**Conclusion:**

Patients dislocating after DAA demonstrated a substantially higher prevalence of adverse spinopelvic mobility despite similar implant orientation and hip restoration. These findings suggest that dynamic pelvic behavior may contribute to anterior instability patterns and highlight the potential relevance of hip–spine assessment in patients undergoing anterior-approach THA.

**Trial registration:**

Retrospectively registered, CNIL MR004 2,225,508.

## Background

Total hip arthroplasty (THA) is a highly effective treatment for hip osteoarthritis, but it is not without complications, with dislocation remaining one of the leading causes of revision [1]. Various factors contributing to instability have been identified, particularly implant positioning parameters such as offset restoration, limb length, cup orientation, and head size [2, 3]. Although optimizing these factors has helped reduce the incidence of dislocation, rates remain between 0.5% and 2% in the literature [1].

Assessment of the hip–spine relationship has enabled patient-specific safe zones to be defined and has highlighted several spinopelvic risk factors for dislocation, including lumbar stiffness (lumbar flexion (LF) ≤ 20°), a standing spinopelvic tilt (SPT) ≤  − 10°, and sagittal imbalance (pelvic incidence (PI) − lumbar lordosis (LL) ≥ 10°) [4]. These abnormalities may cause excessive anterior pelvic rotation (> 20°) [5] when moving from standing to flexed sitting, increasing the risk of posterior dislocation, or excessive posterior rotation (> 10°) when moving from supine to standing, increasing the risk of anterior dislocation. Anticipating such adverse spinopelvic mobility has improved implant positioning strategies [6, 7]; however, most research has focused on the posterior approach, where dislocation risk has traditionally been higher.

Nevertheless, instability also occurs after the direct anterior approach (DAA) [8], and an association with unfavorable lumbopelvic kinematics has been suggested. In an era of increasingly personalized arthroplasty, regarding both implant selection and functional positioning, the choice of surgical approach according to patient phenotype may become relevant. Yet no study to date has compared the prevalence of adverse spinopelvic mobility in dislocations according to surgical approach.

This study aimed to compare two dislocation cohorts, one following a posterior approach and one following a DAA, using post-operative functional imaging and CT analysis to evaluate the presence of adverse spinopelvic mobility and related risk factors. A secondary aim was to assess anatomical and implant-positioning parameters in both groups.

## Methods

### Study design and participants

A retrospective series of 133 patients who experienced THA dislocation and had available post-operative lateral functional radiographs and low-dose CT scans was included. Imaging was performed following the dislocation event and reduction, at the time of instability assessment, between March 2021 and September 2023. The cohort comprised 85 dislocations after a posterior approach (63.9%) and 48 after a direct anterior approach (36.1%). Of the 133 included patients, 28 originated from our institution and 105 from the Corin Registry (OPSReView platform). Although originating from different settings, all patients met the same predefined eligibility criteria and were analyzed using an identical radiographic and spinopelvic assessment protocol. Imaging acquisition followed standardized protocols across centers, although minor inter-center variability cannot be excluded. 86 patients were operated on in Australia (64.7%), 33 in France (24.8%), 11 in the United States (8.3%), and 3 in England (2.3%). The mean age of the patients was 63.6 years (27–88 years), with 81 women and 52 men. All consecutive patients who experienced dislocation after primary THA were included. No patients were excluded. For registry-derived cases, certain peri-operative variables, including the exact date of the index THA, the direction of dislocation, and detailed implant model information, were not consistently available. Therefore, these parameters were not included in the analysis to ensure homogeneity of the pooled dataset. This study was approved by the local Ethics Committee.

Two lateral X-rays were taken for each patient, one of the upper body, standing in a relaxed posture with their feet shoulder-width apart, and one in a flexed-seated position, with their femurs parallel to the floor.

### Spinopelvic and pelvic mobility parameters

**Fig. 1:**
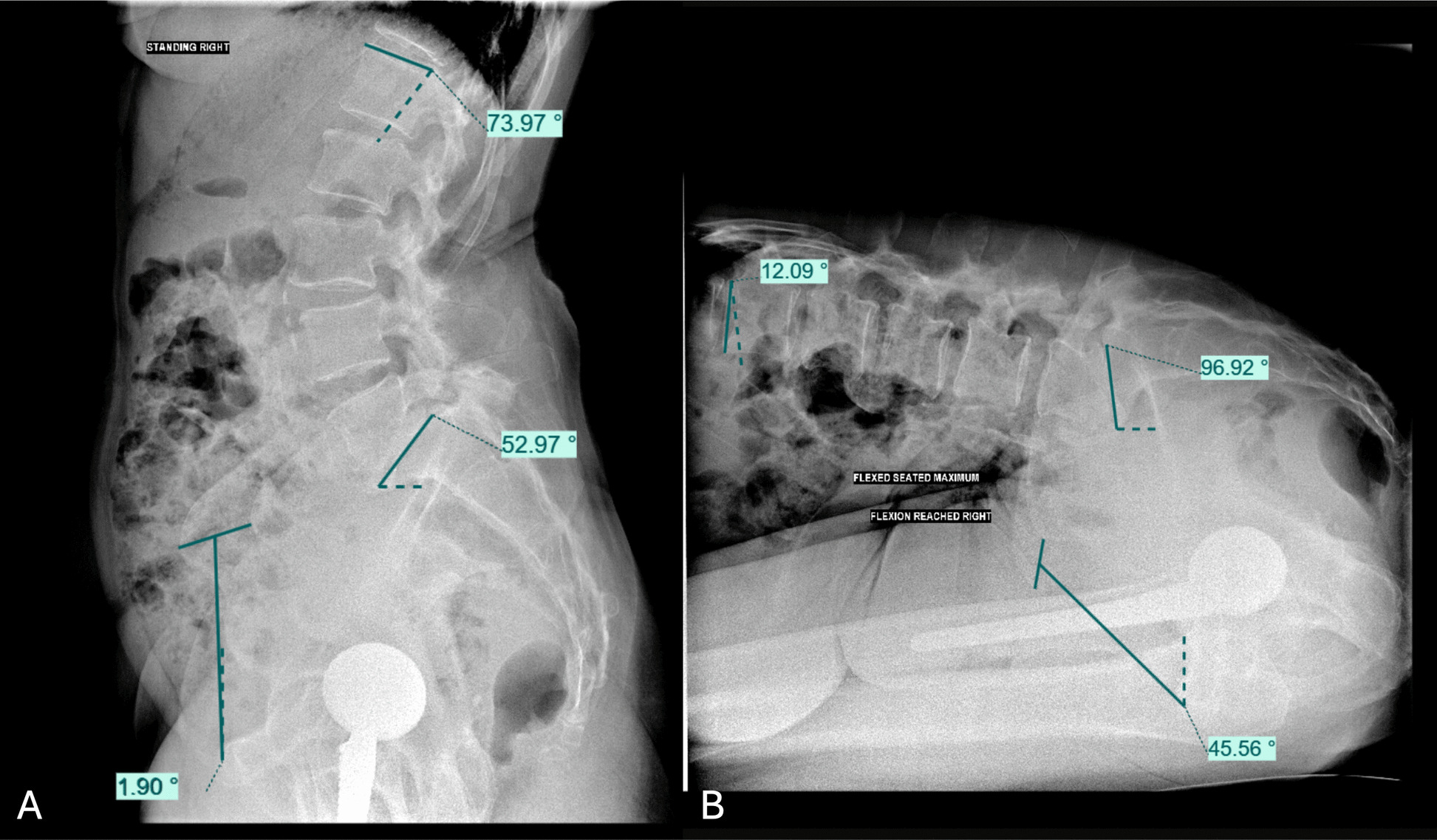
Measurement of spinopelvic parameters on functional lateral radiographs. Standing (**A**) and flexed-seated (**B**) lateral radiographs illustrating spinopelvic measurements. In this example, lumbar lordosis decreased from 73.97° in standing to 12.09° in the seated position, corresponding to a lumbar flexion of 61.9°. Spinopelvic tilt increased from 1.09° to 45.56°, resulting in an ∆SPT of 44° between standing and seated positions

The measurements taken on the lateral X-rays were sacral slope (SS), standing and flexed-seated lumbar lordosis (LL), and standing and flexed-seated SPT. Anterior rotation of SPT was assigned a positive value, and posterior rotation of SPT was assigned a negative value. An increase in SPT denotes an anterior rotation of the pelvis that is equivalent to anteversion, which decreases PT (Fig. 1). The measurements taken from the bony landmarks on the CT scan were the PI.

We investigated the PI-LL mismatch, defined as the difference between PI and LL in the standing position, and the LF, defined as the difference between standing and flexed-seated LL.

The parameters included pelvic mobility during transition from the standing to sitting position, measured as the difference between standing and flexed-seated SPT (∆SPT), and from the supine to the standing position.

Due to the retrospective registry-based design, not all imaging parameters were systematically collected at the time of the initial assessment. Therefore, denominators vary depending on the availability of specific radiographic or CT measurements.

All imaging findings were analyzed by two independent engineers using standardized protocols; however, assessors were not formally blinded to surgical approach due to the clinical context of registry-based evaluations.

### Radiographic evaluation of component alignment and hip restoration parameters

The measurements taken on the CT scan included the hip length as the perpendicular distance from the trans-teardrop line to the superior lesser trochanters when projected onto the supine coronal plane, and we analyzed the hip length difference (HLD) [9] between the hips.

The global offset is the sum of the femoral offset (distance from the proximal femur axis to the femoral head center) and the acetabular offset (distance from the femoral head center to a line passing through the teardrop and perpendicular to the trans-teardrop line). We analyzed the difference in global offset between the two hips.

Finally, for each patient, we measured the cup inclination and anteversion, the stem version, and the combined anteversion.

### Outcome

The outcome of interest was adverse spinopelvic mobility, defined as a change in sacral slope (∆SPT) ≥ 20° between the standing and relaxed-sitting positions [5–8], or ≤  − 13° between the supine and standing positions [4, 8]. We analyzed the spinopelvic risk factors: SPT ≤  − 10°, LF ≤ 20°, PI-LL ≥ 10°, and PI ≤ 41°.

### Statistical analyses

Continuous variables are described as means and ranges. Normality and heteroskedasticity of data were assessed with the Shapiro–Wilk test and Levene’s test. We compared means and proportions between these groups by using Student’s *t* tests, analyses of variances (Mann–Whitney tests), or *chi*-squared tests (or Fisher’s exact tests if appropriate).

A multivariable logistic regression analysis was performed to assess whether the surgical approach remained associated with adverse spinopelvic mobility after adjustment for implant-related parameters. Femoral head diameter was modeled as a continuous variable to avoid arbitrary threshold effects, and global offset was included as a covariate.

All analyses were performed using R (version 4.0.0, R Foundation for Statistical Computing, Vienna, Austria) and EasyMedStat (version 4.2, Paris, France).

## Results

### Analysis of spinopelvic parameters between the two cohorts

Spinopelvic parameters were largely comparable between the posterior approach (PA) and direct anterior approach (DAA) groups. Standing SPT, lumbar flexion, pelvic incidence, PI-LL mismatch, and lumbar lordosis showed no significant differences.

In contrast, the change in SPT from standing to seated position was smaller in the PA group (4.8°, range − 42.1° to 36.7°) compared with the DAA group (13.6°, range − 25.6° to 47.1°) (*p* = 0.007). Similarly, the change in SPT from supine to standing was − 6.4° (range − 14.0° to 11.3°) in the PA group and − 8.5° (range − 16.0° to 5.4°) in the DAA group, with a significant difference between groups (*p* = 0.009). (Table 1).

**Table 1:** Spinopelvic and pelvic mobility parameters

	**Posterior approach**	**Direct anterior approach**	***p* value**
	*n* = 85	*n* = 48	
Spinopelvic parameter			
SPT standing, mean (range)	− 5.2° (− 19.5/14.3)	− 4.7° (− 26.6/17.3)	0.78
Lumbar flexion, mean (range)	38.1° (0.1/69.1)	40.5° (4.1/86.0)	0.437
Pelvic incidence, mean (range)	55.4° (35.4/87.2)	56.1° (36.8/92.2)	0.885
PI-LL, mean (range)	2.9° (− 23.4/36.0)	5.7° (− 22.0/51.3)	0.265
Lumbar lordosis, mean (range)	53.2° (20.8/81.2)	50.7° (20.2/86.2)	0.307
∆SPT standing to seated, mean (range)	4.8° (− 42.1/36.7)	13.6° (− 25.6/47.1)	**0.007**
∆SPT supine to standing, mean (range)	− 6.4° (− 14.0/11.3)	− 8.5° (− 16.0/5.4)	**0.009**

Abbreviations: LL: lumbar lordosis; PI: pelvic incidence; SPT: spinopelvic tilt

Bold values denote statistical significance at the *p* < 0.05 level

### Risk factors associated with dislocation according to the initial approach

Regarding spinopelvic risk factors, most parameters showed no significant difference between the posterior approach (PA) and the direct anterior approach (DAA). The proportion of patients with a standing SPT ≤  − 10° was similar between groups (26.9% in PA vs. 32.6% in DAA, *p* = 0.639). No patient in either group had lumbar flexion ≤ 20°, and low pelvic incidence (≤ 41°) was uncommon in both groups (5.8% vs. 4.4%, *p* = 0.999). Likewise, the proportion of patients with PI-LL mismatch ≥ 10° did not differ significantly (24.6% vs. 33.1%, *p* = 0.593).

**Fig. 2:**
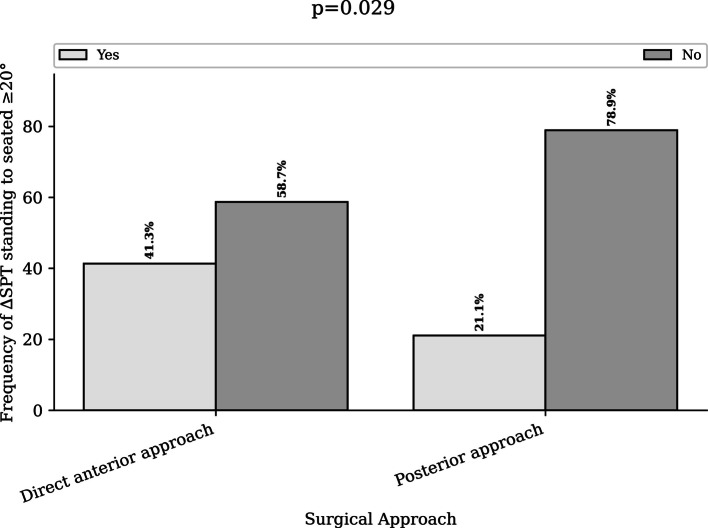
Distribution of patients with adverse spinopelvic mobility (ΔSPT ≥ 20°) from standing to flexed seated according to surgical approach

**Fig. 3:**
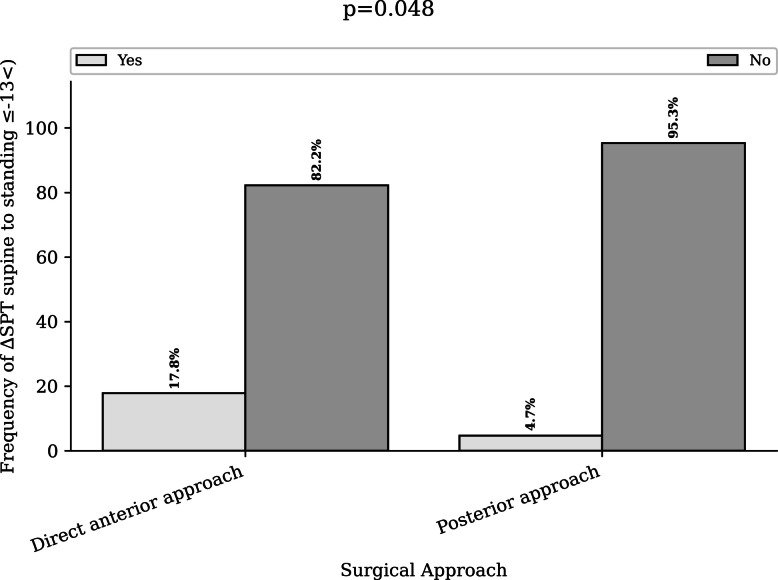
Distribution of patients with adverse spinopelvic mobility (ΔSPT ≤  − 13°) from supine to standing position by surgical approach

In contrast, a ∆SPT ≥ 20° from standing to seated occurred more frequently in the DAA group (41.3%) than in the PA group (21.1%) (*p* = 0.029) (Fig. 2). Similarly, a ∆SPT ≤  − 13° from supine to standing was more common in the DAA group (17.8%) compared with the PA group (4.7%) (*p* = 0.0*48*). (Table 2 and Fig. 3).

**Table 2:** Risk factors associated with dislocation according to the initial approach

	**Posterior approach**	**Direct anterior approach**	***p* value**
	*n* = 85	*n* = 48	
Spinopelvic parameter risk factor			
SPT ≤ − 10°, no. (%)	21/78 (26.9%)	15/46 (32.6%)	0.639
Lumbar flexion ≤ 20°, no. (%)	0/76 (0%)	0/46 (0%)	NC
Pelvic incidence ≤ 41°, no. (%)	4/69 (5.8%)	2/45 (4.4%)	0.999
PI-LL ≥ 10°, no. (%)	16/65 (24.6%)	14/45 (31.1%)	0.593
∆SPT ≥ 20° standing to seated, no. (%)	16/76 (21.1%)	19/46 (41.3%)	**0.029**
∆SPT ≤ − 13° supine to standing, no. (%)	3/64 (4.7%)	8/45 (17.8%)	**0.048**

Denominators vary depending on the availability of radiographic measurements for each parameter

Abbreviations: LL: lumbar lordosis; PI: pelvic incidence; SPT: spinopelvic tilt; NC: not calculable

Bold values denote statistical significance at the *p* < 0.05 level

### Component alignment and hip restoration parameters

Post-operative radiographic parameters were mostly similar between the posterior approach (PA) and direct anterior approach (DAA) groups. HLD did not differ significantly, either when analyzed as a continuous variable (1.3 mm in PA vs. − 0.3 mm in DAA, *p* = 0.147) or when using the threshold of <  − 5 mm (11.6% vs. 15.5%, *p* = 0.579). Cup inclination and stem anteversion were also comparable between groups (*p* = 0.295 and *p* = 0.370, respectively), as was combined anteversion (CA) (*p* = 0.386).

In contrast, two parameters differed between approaches. Offset restoration was significantly more affected in the PA group, with a mean offset difference of 2.8 mm compared with − 0.9 mm in the DAA group (*p* < 0.001). Additionally, cup anteversion was slightly higher in the PA group (23.6° vs. 20.6°, *p* = 0.037). (Table 3).

**Table 3:** Component alignment and hip restoration parameters according to the initial approach

	**Dislocation cohort**		
	**Posterior approach** ***n* = 85**	**Direct anterior approach** ***n* = 48**	***p* value**
Postoperative measurement			
HLD (mm), mean (range)	1.3 (− 12.3/40.9)	− 0.3 (− 11.0/11.0)	0.147
HLD < − 5 mm, no. (%)	8/69 (11.6%)	7/45 (15.5%)	0.579
Offset difference (mm), mean (SD)	2.8 (± 4.34)	− 0.9 (± 4.14)	** < 0.001**
Cup inclination, mean (range)	41.7° (20.7/63.3°)	41.1° (28.7/61.3)	0.295
Cup anteversion, mean (range)	23.6° (4.52/42)	20.6° (5.0/40.4)	**0.037**
Stem anteversion, mean (range)	14.6° (− 6.8/36.8)	16.3° (− 4.9/47.1)	0.37
Combined anteversion, mean (range)	37.9° (8.8/74.7)	36.9° (7.0/75.7)	0.386
Head sizes, mean (SD)	35.95 (± 2.84)	34.04 (± 2.39)	** < 0.001**

Abbreviations: HLD: Hip length difference

Bold values denote statistical significance at the *p* < 0.05 level

Femoral head diameter distribution differed between groups. In the DAA cohort (*n* = 48), 50% of patients received a 32-mm head and 43.8% a 36-mm head, with very limited use of larger diameters. In the posterior approach cohort (*n* = 85), 60% received a 36-mm head and 14.1% a 40-mm head, whereas 32-mm heads accounted for 21.2% of cases.

In multivariable analysis including surgical approach, femoral head diameter, and global offset, the direct anterior approach remained independently associated with adverse spinopelvic mobility (OR 3.09, 95% CI, 1.14–8.34, *p* = 0.026). Neither head diameter (*p* = 0.217) nor global offset (*p* = 0.928) was significantly associated with mobility patterns. At equivalent head size and offset, the difference in dynamic pelvic motion between approaches persisted.

## Discussion

The assessment of the hip–spine relationship has become an essential component of pre-operative planning for total hip arthroplasty [10]. Identifying adverse spinopelvic mobility is crucial for determining which patients may require adjusted component orientation to mitigate the risks of impingement and dislocation [7]. Although dislocation has historically been reported more frequently following the posterior approach, the present findings raise the question of whether functional analysis should also be considered prior to surgery performed through the anterior approach.

In this study, adverse spinopelvic mobility was found to be twice as common in patients who sustained a dislocation after a direct anterior approach. Four in ten patients demonstrated abnormal pelvic movement when transitioning from standing to a flexed seated position, and nearly one in five when moving from supine to standing. By contrast, the prevalence of established spinopelvic risk factors did not differ significantly between approaches. Post-operative component positioning was also broadly comparable between groups. These observations underline the importance of considering spinopelvic mobility when attempting to reduce the risk of dislocation following anterior-approach arthroplasty.

### Spinopelvic mobility and risk factors

Spinopelvic risk factors associated with instability and impingement are well recognized [5, 11], and their identification assists in determining which patients may benefit from adjusted component placement. In this cohort, these factors were similarly distributed between approaches. An SPT ≤  − 10° and a sagittal imbalance (PI-LL ≥ 10°) were each present in more than a quarter of patients, with no significant difference between groups. Severe lumbar stiffness was absent in both groups, as no patient exhibited lumbar flexion below 20°, which is consistent with the low prevalence, approximately 2%, reported in population studies [7].

However, significant differences in dynamic spinopelvic behavior were observed. Variations in pelvic tilt of this magnitude may lead to meaningful changes in functional cup orientation and may influence impingement and instability risk. The DAA group demonstrated greater posterior pelvic rotation from supine to standing, with a fourfold higher incidence of ∆SPT ≤  − 13°. This finding is consistent with the predominance of anterior dislocations after DAA [8], often resulting from posterior impingement leading to anterior instability. It is therefore unsurprising that this factor was rarely observed in patients who experienced posterior dislocation after a posterior approach, fewer than 5%.

Although posterior dislocation remains more common following the posterior approach, anterior pelvic rotation was more pronounced after DAA, with a mean of 13.6° compared with 4.8° in the PA group. Furthermore, excessive mobility (∆SPT ≥ 20°) was observed in 40% of DAA dislocations, twice the rate seen after PA. While both spinopelvic risk factors and adverse mobility have been associated with instability after both approaches [5, 8], the present results suggest that the hip–spine relationship may play a more prominent role in anterior-approach dislocations, whereas posterior-approach dislocations appear more multifactorial.

Although dislocation following the anterior approach is generally considered less frequent, and despite the majority of literature on spinopelvic mobility being focused on the posterior approach, our findings indicate that functional spinopelvic assessment may in fact be more critical in the context of DAA. Such evaluation may help substantially reduce the risk of dislocation, either by adjusting component orientation according to functional combined anteversion [6, 11, 12] or by selecting implants with intrinsically lower instability risk, such as dual mobility cups [13, 14].

### Component alignment and hip restoration parameters

In this study, implant orientation parameters did not differ significantly between the two groups, with similar femoral version and comparable cup inclination. Only cup anteversion was lower in the DAA group by approximately 3°, which would theoretically reduce the risk of anterior dislocation, although values in both groups remained within commonly recommended ranges (around 20° of anteversion and 41° of inclination). Conversely, combined anteversion was similar between the groups and does not appear to represent a discriminating factor for dislocation risk in either approach. Although combined anteversion has been shown to influence the likelihood of instability [15], excessive CA did not seem to be associated with dislocations after DAA[8], whereas low CA appeared to play a more important role in dislocations following the posterior approach.

Regarding limb length, no significant difference was observed between groups. While limb shortening may increase the risk of instability, length was preserved in the DAA group and increased by an average of 1 mm in the PA group, making a substantial confounding effect unlikely [16]. The only parameter that differed between groups was global offset, which was reduced by 0.9 mm in the DAA group and increased by 2.8 mm in the PA group. However, when comparing groups, the net intergroup difference approached 4 mm. While reduced offset may decrease soft tissue tension and potentially contribute to instability, particularly in anterior dislocations, the magnitude of change within the DAA cohort itself was modest. Previous studies have reported that small variations in joint center reconstruction may fall within the range of post-operative measurement variability [17]. Although implant-related parameters differed between groups and may have contributed to the occurrence of instability, the difference in spinopelvic mobility between approaches persisted after adjustment for head diameter and offset, although differences in femoral head diameter may still represent a potential confounding factor. This suggests that implant selection alone does not account for the observed dynamic differences.

Overall, implant orientation and restoration of native hip parameters are well-recognized contributors to dislocation risk [18], yet they differed only minimally between the two groups in this cohort, and a recent study showed that operative changes in leg length and offset did not demonstrate any clinically or statistically significant effect on the dislocation risk [19].

### Strengths and limitations

This study has several strengths. It represents one of the largest cohorts of dislocating THA patients in whom functional lateral radiographs and low-dose CT scans were both available, allowing a detailed assessment of spinopelvic morphology, dynamic pelvic motion, and implant positioning. The comparison of dislocations following both the posterior and anterior approaches provides new information in an area with scarce data, and all imaging analyses were performed by independent assessors using standardized protocols.

However, several limitations must be acknowledged. First, the study is retrospective. This may introduce selection bias; however, the objective imaging data used for all measurements are unaffected by retrospective design, and the analysis does not rely on patient recall or subjective reporting. Second, the multicenter design may introduce variability in surgical technique or implant selection. Yet this heterogeneity reflects real-world practice and increases external validity, and the consistent patterns observed across centers indicate that the key findings are not driven by center-specific effects. Third, only patients who dislocated were included, without a non-dislocating control group. This prevents estimation of absolute risk, but it does not compromise the primary aim, which was specifically to compare the patterns of adverse spinopelvic mobility between approaches among patients who actually experienced instability. Because imaging was performed after the dislocation event and the exact timing was not systematically documented, spinopelvic mechanics may have been influenced by pain, apprehension, or adaptive guarding. Consequently, the observed mobility patterns may not fully reflect the pre-dislocation biomechanical state. Moreover, the registry-based nature of a large portion of the cohort limited access to certain perioperative variables, including the timing of the index arthroplasty, the documented direction of instability, and detailed implant model characteristics. Because inclusion required the availability of complete functional imaging, patients included in the registry may not represent all dislocation cases during the study period, which may introduce a degree of selection bias. In particular, the absence of consistent information regarding the direction of dislocation (anterior versus posterior) limits the ability to directly correlate dynamic spinopelvic patterns with specific instability mechanisms.

In addition, not all imaging parameters were systematically recorded in the original registry dataset, resulting in variable denominators across analyses. While no patients were excluded based on clinical characteristics, incomplete measurement availability represents a limitation inherent to retrospective registry-derived data. These factors do not affect the comparative assessment of spinopelvic mobility between approaches but restrict interpretation regarding temporal patterns of instability and specific implant-related risk factors. Finally, some subgroup analyses involved relatively small sample sizes, which may increase the risk of type II error. Nonetheless, the differences observed in dynamic pelvic motion were large and statistically significant, making it unlikely that the main findings are due to insufficient sample size.

## Conclusions

In this multicenter cohort of patients with THA dislocation, spinopelvic morphology was similar between posterior and anterior approaches, whereas dynamic spinopelvic mobility differed substantially. Adverse spinopelvic mobility was more frequent in anterior-approach dislocations, particularly during transitions from standing to seated and from supine to standing, suggesting a distinct instability mechanism. Although causality cannot be established without a non-dislocating control group, these findings highlight the potential relevance of functional hip–spine assessment in understanding anterior instability patterns.

## Data Availability

The datasets used and/or analyzed during the current study are available from the corresponding author on reasonable request.

## Abbreviations

THA: Total hip arthroplasty
PI: Pelvic incidence
LL: Lumbar lordosis
SPT: Spinopelvic tilt
LF: Lumbar flexion
SS: Sacral slope
CA: Combined anteversion
HLD: Hip length difference
DAA: Direct anterior approach
